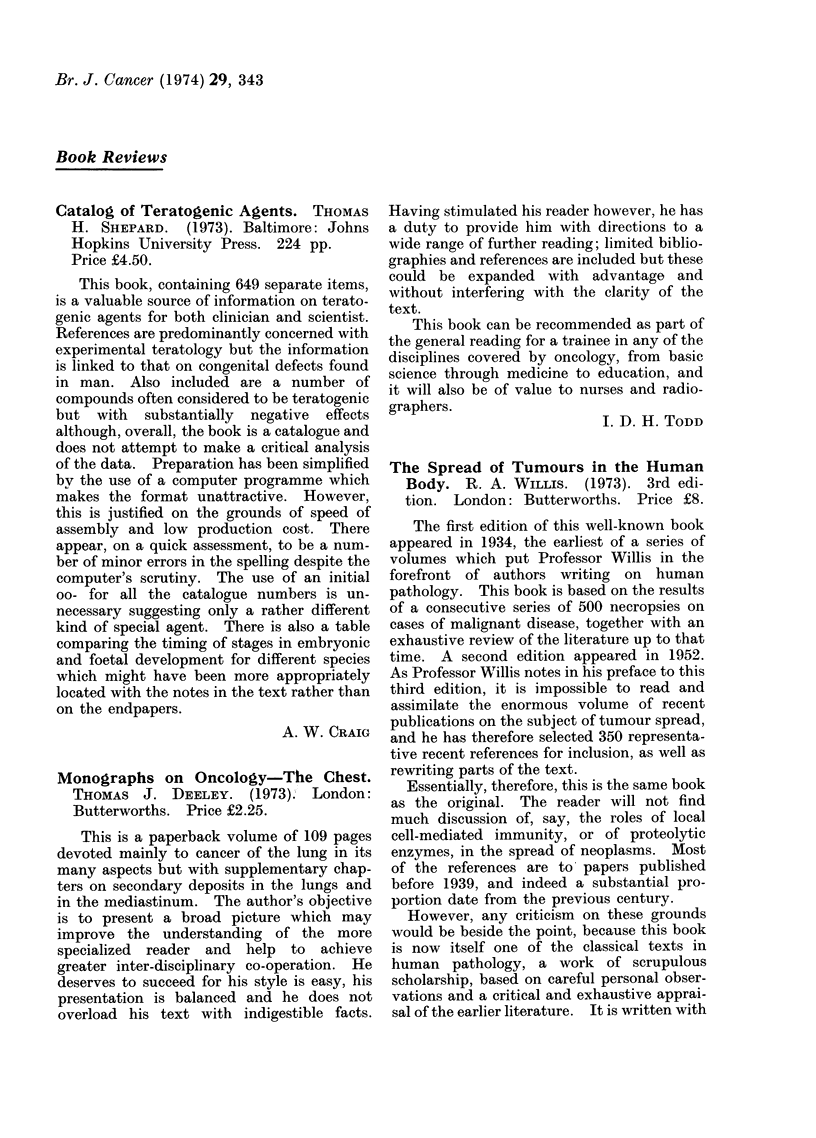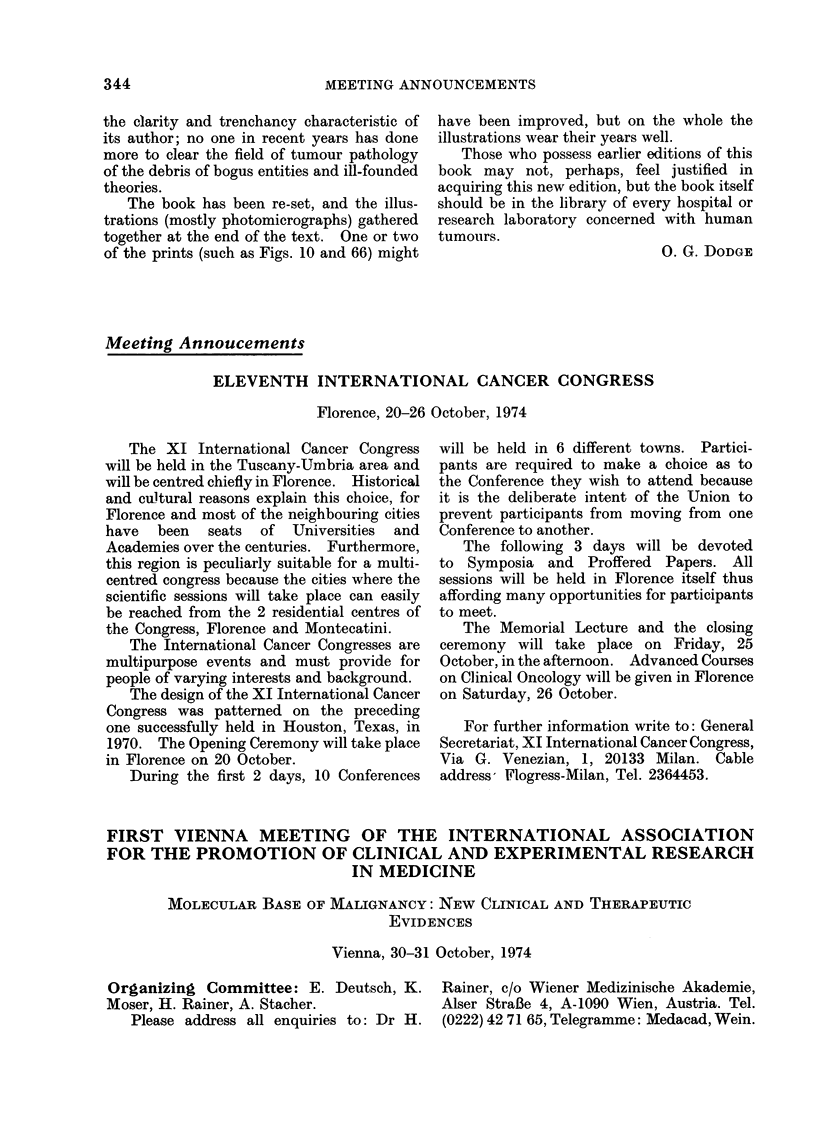# The Spread of Tumours in the Human Body

**Published:** 1974-04

**Authors:** O. G. Dodge


**The Spread of Tumours in the Human Body.** R. A. WILLIS. (1973). 3rd edition. London: Butterworths. Price £8.

The first edition of this well-known book appeared in 1934, the earliest of a series of volumes which put Professor Willis in the forefront of authors writing on human pathology. This book is based on the results of a consecutive series of 500 necropsies on cases of malignant disease, together with an exhaustive review of the literature up to that time. A second edition appeared in 1952. As Professor Willis notes in his preface to this third edition, it is impossible to read and assimilate the enormous volume of recent publications on the subject of tumour spread, and he has therefore selected 350 representative recent references for inclusion, as well as rewriting parts of the text.

Essentially, therefore, this is the same book as the original. The reader will not find much discussion of, say, the roles of local cell-mediated immunity, or of proteolytic enzymes, in the spread of neoplasms. Most of the references are to papers published before 1939, and indeed a substantial proportion date from the previous century.

However, any criticism on these grounds would be beside the point, because this book is now itself one of the classical texts in human pathology, a work of scrupulous scholarship, based on careful personal observations and a critical and exhaustive appraisal of the earlier literature. It is written with the clarity and trenchancy characteristic of its author; no one in recent years has done more to clear the field of tumour pathology of the debris of bogus entities and ill-founded theories.

The book has been re-set, and the illustrations (mostly photomicrographs) gathered together at the end of the text. One or two of the prints (such as Figs. 10 and 66) might have been improved, but on the whole the illustrations wear their years well.

Those who possess earlier editions of this book may not, perhaps, feel justified in acquiring this new edition, but the book itself should be in the library of every hospital or research laboratory concerned with human tumours.

O. G. DODGE